# Supplementary material for: Impact of Remote Ischemic Preconditioning Conducted in Living Kidney Donors on Renal Function in Donors and Recipients Following Living Donor Kidney Transplantation: A Randomized Clinical Trial
Source: J Clin Med. 2019 May 20;8(5):713. doi: 10.3390/jcm8050713 (PMC6572316; doi:10.3390/jcm8050713)
Supplement: Supplementary file 1 [file jcm-08-00713-s001.pdf]

Supplementary

Table S1. Comparison of perioperative characteristics of kidney donor and recipients according to gender.

	Female			Male		
	**Control (*n* = 39)**	**RIPC (*n* = 59)**	***p*-Value**	**Control (*n* = 46)**	**RIPC (*n* = 26)**	***p*-Value**
Age, year	45.2 ± 8.7	44.1 ± 9.0	0.54	38.3 ± 12.6	41.7 ± 9.1	0.24
ASA PS 1/2	28/11	43/16	1.00	32/14	16/10	0.66
Weight	57.3 ± 8.1	58.7 ± 7.8	0.41	73.4 ± 10.1	72.3 ± 10.1	0.66
Height	157.9 ± 6.2	158.5 ± 5.7	0.64	171.4 ± 5.6	171.8 ± 6.5	0.79
Body mass index, kg/m^2	23.0 ± 2.7	23.4 ± 2.9	0.50	24.9 ± 2.6	24.4 ± 2.7	0.45
Preoperative hemoglobin	12.9 ±0.9	13.1 ± 1.0	0.58	14.9 ± 1.0	14.9 ± 1.0	0.79
Preoperative serum creatinine, mg/dL	0.7 (0.6–0.7)	0.6 (0.6–0.7)	0.60	0.9 (0.8–1.0)	0.9 (0.8–1.0)	0.79
Preoperative, eGFR, mL/min/1.73 m^2	106.3 ± 7.7	108.0 ± 10.5	0.35	106.8 ± 13.5	104.1 ± 10.6	0.37
Operation time	205.9 ± 28.3	202.9 ± 39.8	0.61	225.0 ± 39.8	216.2 ± 37.4	0.36
Operation site (right), *n* (%)	14 (35.9)	28 (47.5)	0.36	20 (43.5)	8 (30.80	0.42
Urine output, mL	324.1 ± 168.1	307.9 ± 159.3	0.63	329.1 ± 159.5	291.5 ±127.8	0.31
Mean blood pressure, mmHg						
Before RIPC	80.7 ± 10.2	79.6 ± 12.2	0.62	84.5 ± 12.4	79.2 ± 10.6	0.07
After RIPC	83.0 ± 9.1	84.0 ± 12.1	0.67	85.2 ± 10.0	88.5 ± 14.1	0.30
Cystatin C, mg/L	0.7 (0.6–0.8)	0.6 (0.6–0.7)	0.02	0.8 (0.7–0.9)	0.8 (0.8–0.8)	0.76

RIPC, remote ischemic preconditioning; ASA PS, American Society of Anesthesiologists physical status; eGFR, estimated glomerular filtration rate based on the CKD-EPI equation. Data are expressed as mean ± SD, median and interquartile range, or number (%), as appropriate.

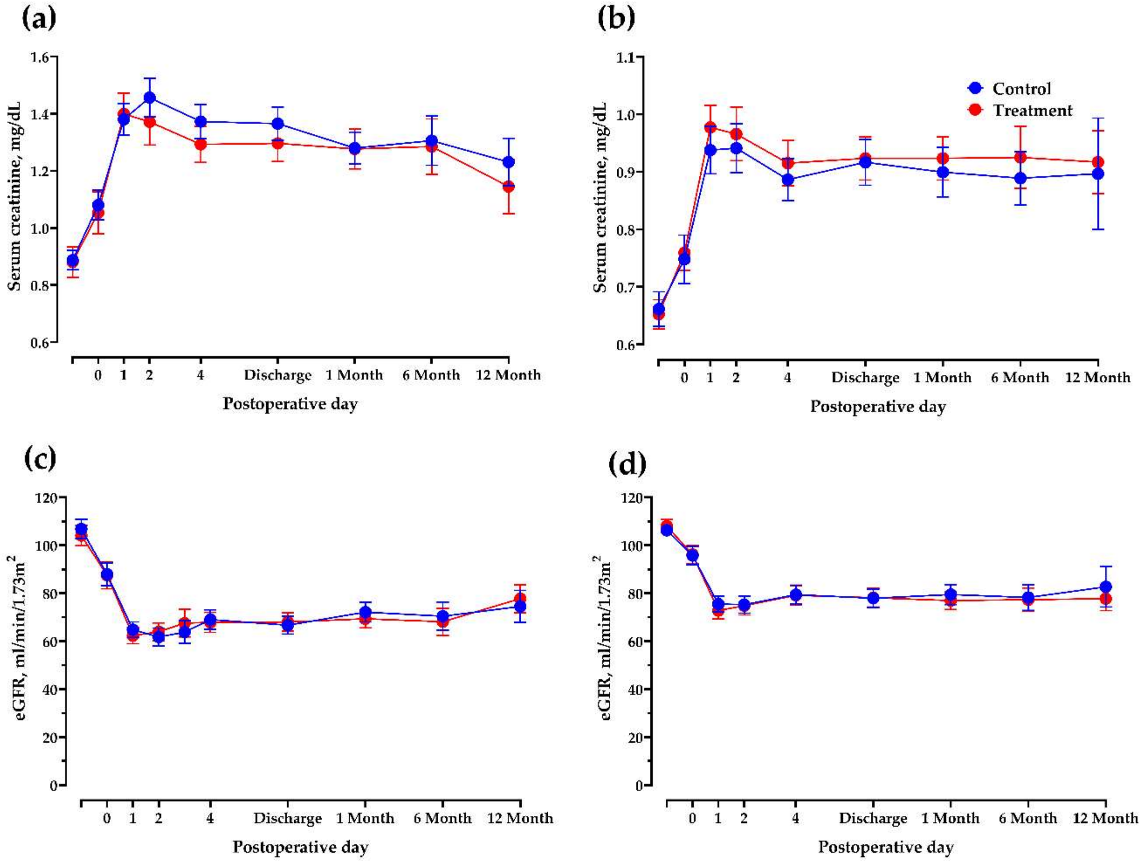

Figure S1. Serial changes in serum creatinine concentration and estimated glomerular filtration rate in male and female kidney transplant donors for 1 year after transplantation: (**a**) Among male donors, postoperative serum creatinine (sCr) concentrations were not significantly different in the control and the RIPC group during the postoperative period. (**b**) Among female donors, sCr concentration did not differ in the RIPC and control groups at any time point. (**c**) Among male donors, estimated glomerular filtration rate (eGFR) was calculated using the CKD-EPI equation. Among male donors, eGFR did not differ between RIPC and control groups at any time point. (**d**) Among female donors, estimated glomerular filtration rate (eGFR) was calculated using the CKD-EPI equation. eGFR did not differ between RIPC and control groups at any time point.
